# Multiple invasive species affect germination, growth, and photosynthesis of native weeds and crops in experiments

**DOI:** 10.1038/s41598-023-48421-w

**Published:** 2023-12-13

**Authors:** Magdalena Lenda, Bastian Steudel, Piotr Skórka, Zuzanna B. Zagrodzka, Dawid Moroń, Renata Bączek-Kwinta, Franciszek Janowiak, Agnieszka Baran, Hugh P. Possingham, Johannes M. H. Knops

**Affiliations:** 1 School of Biological Sciences, The University of Queensland, Brisbane, QLD Australia; 2 Institute of Nature Conservation, Polish Academy of Sciences, Mickiewicza 33, 31-120 Kraków, Poland; 3 Department of Health and Environmental Sciences, School of Science, Xi’an Jiaotong-Liverpool University, Suzhou, 215123 Jiangsu China; 4 School of Biosciences, The University of Sheffield, Sheffield, UK; 5 Institute of Systematics and Evolution of Animals, Polish Academy of Sciences, Sławkowska 17, 31-016 Kraków, Poland; 6 Department of Plant Breeding, Physiology, and Seed Science, Faculty of Agriculture and Economics, University of Agriculture in Cracow, Podłużna 3, 30-239 Kraków, Poland; 7 The Franciszek Górski Institute of Plant Physiology, Polish Academy of Sciences, Niezapominajek 21, 30-239 Kraków, Poland; 8 Department of Agricultural and Environmental Chemistry, Faculty of Agriculture and Economics, University of Agriculture in Cracow, Al. Mickiewicza 21, 31-120 Kraków, Poland

**Keywords:** Ecology, Plant sciences, Ecology, Environmental sciences

## Abstract

Alien plant species regularly and simultaneously invade agricultural landscapes and ecosystems; however, the effects of co-invasion on crop production and native biodiversity have rarely been studied. Secondary metabolites produced by alien plants may be allelopathic; if they enter the soil, they may be transported by agricultural activities, negatively affecting crop yield and biodiversity. It is unknown whether substances from different alien species in combination have a greater impact on crops and wild plants than if they are from only one of the alien species. In this study, we used a set of common garden experiments to test the hypothesis that mixed extracts from two common invasive species have synergistic effects on crops and weeds (defined as all non-crop plants) in European agricultural fields compared to single-species extracts. We found that both the combined and individual extracts had detrimental effects on the seed germination, seedling growth, biomass, and photosynthetic performance of both crops and weeds. We found that the negative effect of mixed extracts was not additive and that crop plants were more strongly affected by invasive species extracts than the weeds. Our results are important for managing invasive species in unique ecosystems on agricultural land and preventing economic losses in yield production.

## Introduction

During the Anthropocene, invasive alien species (hereafter referred as ‘invasive species’) spread across the planet and have caused increasingly larger ecological and environmental impacts, causing biodiversity decline and species extinctions^1,2^. Invasive species rarely colonize new habitats alone; there are at least 5789 naturalized alien plant species in Europe^3^, and invaded plant communities are frequently dominated by several invasive species^4^. In plant communities dominated by invasive species, the latter interact with each other, and their combined impact on both the environment and native species is important^4,5^. The combined impact of multiple invasive species may be (1) simply a summation of the effects, (2) synergistic (i.e. more than the summation of effects), (3) less than the summation of effects, or (4) less than the individual impact of each species^4,6^. The last effect may occur if invasive species compete, thereby reducing their combined effect^4^.

Currently, 37% of Earth’s land is used for agricultural purposes, with approximately 11% used for growing crops and the remainder for pastures^7^. Thus, the biodiversity of cultivated land plays an important role in global biodiversity^8,9^. Along with crop species, agricultural lands also contain a diverse array of native species that are important components of the total biodiversity, especially pollinators, as weeds can be a food source when other grassland flowers are unavailable^10^. However, research examining plant invasions has largely focused on natural habitats, whereas farmland invasions are poorly understood^1^. In Europe, cultivated land comprises a mosaic of crop fields, grasslands, meadows, pastures, abandoned fields, hedgerows, field margins, buffer strips, and human settlements^11,12^. This has created unique cultural landscapes rich in biodiversity^11,12^ and threatened species^11,13^. In Central and Eastern Europe, many agricultural lands were abandoned after the collapse of communism in the late 1980s, and these abandoned lands were colonized by invasive plant species that formed monocultures in some ecosystems (Fig. S1)^14,15^. Especially goldenrod tends to form patches of only this invader, while others, including walnut reduce the overall biodiversity, but still occur in species mixtures. In addition to abandoned fields, other marginal habitats, less intensively managed meadows, and croplands are frequently dominated by alien species^4,15,16^.

Two common invasive species in Central and Eastern Europe are the Persian walnut (*Juglans regia* L.) and Canadian goldenrod (*Solidago canadensis* L.)^4,15^. *J. regia* L. (Juglandaceae; syn. common walnut, Persian walnut) originated in the area between the Black Sea Basin, Turkey, Central Asia, and the Himalayas, where it occurs in mixed and deciduous forests^17,18^. Walnut has an attractive taste and fatty seeds; thus, it has been cultivated for centuries outside its natural range, including in Central Europe and North America. In Poland, walnuts were introduced to monasteries during the Middle Ages and grew well under Poland’s climatic and edaphic conditions^19^. Walnut invasion is a new European phenomenon caused by recent political-related land-use changes^15,20^. Goldenrod is a noxious, invasive weed that affects wheat fields in Asia and has been reported to decrease crop yields^16^. Goldenrod, native to North America, has spread since its introduction as an ornamental plant and is now widespread globally; it has been reported in 49 countries and is one of the 100 worst invasive species^21,22^. The main period for Goldenrod’s rhizome growth relevant to flowering shoots in central Europe is between July and September^23^. Goldenrod plants can reproduce in their first year under ideal conditions, but remain vegetative if they are too small to reproduce annually^24^. Individual clones can survive up to a century^24^. Both walnuts and goldenrods have invaded 80% of abandoned farmlands, field margins, and extensively managed croplands in Central Europe (Fig. S2)^4,15^. The high abundance of these invasive species in such areas creates an invasion pool that can spread into other native and agricultural lands^11,14,15^. Thus, both native weeds and crops are exposed to invasive species spilling out of abandoned fields^4,14,25^.

Invasive alien plant species can disrupt native ecosystems and crops due to the introduction of novel allelopathic compounds and their competitive advantage in the absence of natural controls^26,27^. Allelopathy is defined as chemical interaction between plants, including these mediated by microbes^28^. This includes the release of biochemical compounds, often called secondary metabolites, by one plant that inhibit the growth, germination, reproduction, and survival of nearby plant species^29^. Other definitions reduce the possible interactions to those which add plant–produced secondary products to the rhizosphere^28^. Or they stress that allelopathic plants release cytotoxic chemicals into the environment which can increase their ability to compete with surrounding organisms for limited resources^30^. Although this is a controversial subject^31,32^, allelopathy has been widely reported in different plant taxa^32^ and almost all plants have ingredients which are putative allelopathic substances^28^. Invasive alien plant species can also inhibit native plant species and crops by releasing allelopathic chemicals into soil, air, or water^29,33–36^. Native plant communities, in contrast, may have more balanced and ecologically integrated allelopathic interactions that contribute to the stability and diversity of their ecosystems due to common evolutionary history^37^. Competition among plants occurs when they use limited resources such as water, nutrients, light, and space. Competition can be direct (in which plants physically interfere with each other) or indirect (in which plants reduce their resource availability). Thus, allelopathy involves chemical interactions between plants even when competition for essential resources does not occur. Both these mechanisms are important drivers of plant interactions and can influence the composition and structure of plant communities in ecosystems^37^.

The direct negative competitive impact of invasive goldenrods on crop production^35,36^ and biodiversity^4,14^ is well documented. However, less attention has been paid to the negative effects of invasive walnuts because this invasion is a new phenomenon^15^, and all experimental data have come from either cultivated plants or the native region of the walnut^34^. Moreover, the indirect effects of interactions between many alien plant species that invade the same area are usually overlooked^4^. We are unaware of any studies examining whether potential allelopathy caused by multiple invasive species has additive or synergistic effects on crops or native plants. Allelopathic substances are usually present in all plant tissues and can be released into the soil from the roots, fallen leaves, or injured plant tissues. Agricultural fieldwork such as plowing, harrowing, or mulching may facilitate the release of allelopathic substances. Allelopathy is typically studied as a single-species effect on crops^34–36^, and, to the best of our knowledge, its effect on native weeds (occurring in both abandoned and managed fields) has not been examined. Note that we use the term “weed” for all non-crop plants, as these plants are not wanted by farmers as they lead to crop contamination. Both goldenrods and walnuts have been reported to have allelopathic effects on other plant species^34–36,38^. Also, both goldenrod and walnut often co-occur with crops^15,16^ and weeds^4,14^ in agricultural landscapes, and agricultural activities commonly incorporate damage and transportation of both plant tissues into the soil. Lenda et al.^4^ showed that goldenrods affect the biodiversity of native flora growing on abandoned post-agricultural fields more than walnuts. This raises the question of whether goldenrods and walnuts have a negative and similar allelopathic influence on crops or weeds and if so, what is their combined effect?

In this study, we used a set of common garden experiments to examine whether plant raw extract of walnut, goldenrod, or their combination have an allelopathic effect on the germination, growth, and physiology of several representative native weeds^39^ and crops^40^. We selected commonly cultivated crops and weeds in areas where walnut and goldenrod are common invasive species that occur either alone or together^4,15^. We hypothesized that: (1) both invasive species will negatively affect the germination, growth, and photosynthetic performance of native weeds and crops; (2) the goldenrod allelopathic effect will be stronger than that of walnut based on preliminary observations, (3) the combined allelopathic impact of both goldenrod and walnut on the germination, growth, and photosynthetic performance of native weeds and crops will be additive or synergistic; a stronger effect than each species individual impact averaged, (4) weeds are more resistant to allelopathy than crops because they commonly interact with allelopathic invasive species in crops and abandoned fields^4^ and are under strong natural selection pressures via competition with crops and other weeds^41^. Furthermore, weed species have a more diverse genetic pool than crop plants, which undergo selection during the breeding of different cultivars.

## Materials and methods

This study was performed from May to July 2014 in three parts: (1) laboratory experiments with seeds germinated in Petri dishes, (2) greenhouse flowerpot experiments on seed growth, and (3) measurements of plant photosynthetic performance in the flowerpot experiment (Tables S1–S10, Figs. S3–S7). Prior to sowing, the seeds were treated with extracts from wild growing and invasive goldenrod; wild growing and invasive walnut; mixed extracts of walnut and goldenrod; and a control treatment of water. The plant material of the invasive species was randomly collected from randomly chosen invaded abandoned fields. The plant species used in the experiments to study their responses to the invasive plant extracts were purchased from commercial suppliers. We used weed seeds from suppliers because we wanted to ensure seeds’ high viability (90% in commercial plants, according to sellers, confirmed in the control treatment), which could be disturbed on invaded land. In addition, crop seeds were purchased from commercial suppliers, and we wanted to keep the weeds the same to compare both groups.

### Procedures for macerating and preparing extracts

To prepare individual extracts of goldenrod and walnuts we collected leaves from randomly chosen plants and abandoned fields invaded by these species in the suburbs of Cracow City, Poland. Fresh leaves (300 g) were collected in June (early summer) in sunny weather. The leaves were free of molds and other visible pathogens. The abandoned fields were far from industrial areas, and pesticides had not been used there for at least 10 years. Leaves were shredded using a Philips HR7776/90 blender. The shredded tissues were then mixed with 1500 mL of distilled water and subjected to vortex mixing in a dark box at 20 °C for 24 h. For preparing mixed extracts, 150 g of walnut and 150 g of goldenrod in 1500 mL of distilled water were used. We prepared the mixture using both plant materials in the same extraction process rather than mixing the two single extracts together, as both species co-occur frequently; hence, the target plants will be faced with a mixture of both plants and their interacting allelopathic effects. We chose leaves as the material for the extracts to mimic the natural conditions in agricultural landscapes. In most cases, the green parts of young (up to a few months old) goldenrods and walnuts are mixed with the soil during fieldwork and may affect crops or non-crop plants. In such young plants, the roots are much smaller than the green parts. In addition, extracts from the green parts of goldenrods affect other plants more than those from the roots^38^.

### Experiment in Petri dishes

We examined four crop species: *Brassica oleracea*, *Fagopyrum esculentum*, *Lupinus albus*, and *Triticum aestivum,* and four non-crop species (weeds) species: *Campanula patula*, *Coronilla varia*, *Matricaria chamomilla*, and *Trifolium repens.* A disc of blotting paper was placed into each 6 cm diameter Petri dish, and 3 mL^3^ of the respective extract or distilled water (control treatment) was added once. *M. chamomilla* seeds were germinated in scattered natural light due to their requirement for illumination during germination (positive photoblasticity)^42^. Watering once with extracts of goldenrod and/or walnut prepared as described above was used in the experiment because it resembled the most natural conditions – invasive species were damaged once by fieldwork, such as plowing, before crops were sown in agricultural fields, and weeds germinated from seedbanks. We used deionized water to produce extracts to eliminate chemicals that could interact with the plant substances in the extracts. Subsequently, we watered all Petri dishes with 1 mL of filtered tap water every other day. We used different numbers of seeds for different plant species depending on their seed size because we used the same pot size for all species. A total of 1200 seeds were examined, with 300 seeds per treatment and 120–200 seeds per species (Table S1). The Petri dishes were inspected for germination each day. Seeds were considered germinated when the radicles were 1 mm long. The percentage of germinated seeds was calculated for statistical analyses.

### Flowchart of greenhouse flower pot experiment

We examined three crop species, *Fagopyrum esculentum*, *Lupinus albus*, *Triticum aestivum,* and four flowering weed species, *Agrostemma githago*, *Cichorium intybus*, *Matricaria chamomilla*, and *Trifolium repens*. These weed species are important food resources for diverse pollinators in Central European agricultural ecosystems and occur in crops and abandoned fields^4^. For simplicity, we use the term “weed” for all plants that are not crop plants and that are unwanted by farmers. For each species, we used 720–729 seeds (180 seeds per treatment), but for *Cichorium intybus,* we used only 102 seeds because of low seed availability (Table S2). We intended to increase the number of studied species, which is why we used different crops and weed species from those used in the Petri dish experiment. In addition, an insufficient number of seeds were available for *Campanula patula* and *Coronilla varia* for this experiment. Two seeds were sown per flowering pot for *Fagopyrum esculentum* and *Lupinus albus*. For the other species, three seeds were sown per pot. *M. chamomilla* seeds are sown directly onto the soil surface^43^. All the seeds were sown across the diagonal of the pot (upper left, middle, and lower right parts). We used multiple seeds per pot because of greenhouse space limitations. However, this nested design was incorporated into the statistical analyses of the generalized linear mixed models (see below). We used commercially available soil with a content of approximately 250 mg/kg Mg, approximately 300 mg/kg phosphate, approximately 1.4% total nitrogen, approximately 20% organic matter, and a pH of less than 4.5. The Mg content was determined using the Schachtschabel method in 0.0125 mol CaCl2 dm-3, phosphate using the Egner-Rhiem method, nitrogen using the Kjeldahl method, organic matter using the loss on ignition method, and pH using the potentiometric method with 1 mol KCl.

Each pot (5 × 5 × 5 cm) was watered once with 25 mL of the extract (or distilled water for the control treatment). Watering once with extracts was used in the experimental procedure because it resembled the most natural conditions—invasive species are damaged once by fieldwork, such as plowing, before crops are sown in agricultural fields, and weeds germinate from seedbanks. We used deionized water to produce extracts to eliminate chemicals that could interact with the plant substances in the extracts. All pots were watered every other day with 25 mL of filtered tap water. Each flowerpot was inspected daily to record seed germination and determine whether seedlings survived the first 2 weeks after germination (some seedlings died, Table S3). At 2 weeks (first measurement) and 4 weeks (second measurement), seedling length (rounded to the nearest millimeter) and number of leaves were measured (Tables S4 and S5). After 4 weeks, the seedlings were thinned to one (randomly chosen) per pot to assess photosynthetic performance and then removed and weighed to the nearest 0.1 g to determine both above- and below-ground biomass (Table S6). The light conditions in the greenhouse were the natural light conditions with a 14 h day light intensity of about 400–500 μmol photons per square meter and second and filtered using a mono layer of silicate window glass. The temperature was about 20–23 ℃ at day and 17–20 ℃ at night. The relative air humidity was approximately 30%. Under full sun exposure outside 1200–1500 μmol photons per square meter and second can be reached in the climatic zone the experiment was conducted.

### Measuring plant photosynthetic performance

The fluorescence parameters of chlorophyll *a* and the total chlorophyll content were measured spectrophotometrically. The following fluorescence parameters were used: (1) the maximum efficiency of PS II (F_v_/F_m_), (2) photochemical quenching (q_P_), (3) non-photochemical quenching (NPQ), and for chlorophyll content, and (4) greenness index.

The chlorophyll fluorescence parameters were measured using an *FMSII* pulse-amplitude-modulated fiber optic system (Hansatech, Kings Lynn, UK). Leaf clips (with a 5-mm diameter hole) were fastened to the leaves and kept there for 20 min for dark adaptation. Minimal fluorescence in the dark-adapted state (F_0_) was measured, and a saturating-light pulse [10,000 μmol (photon) m^−2^ s^−1^ for 0.9 s] was used to determine maximal fluorescence in the dark-adapted state (F_m_). Next, the leaf was irradiated with actinic light [1500 μmol (photon) m^−2^ s^−1^] for 270 s to measure its steady-state fluorescence (F_t_). Then, the minimal fluorescence yield in the light-adapted state (F_0_') was measured by immediately irradiating the leaf for 3 s with a far-red emitting diode (radiation of about 15 W m^–2^). Next, the saturating-light pulse was used again to determine the maximal fluorescence yield in the light-adapted state (F_m_’). The data were automatically used for the following parameters: the maximum efficiency of PSII photochemistry was calculated as F_v_/F_m_, where F_v_ = F_m_–F_0_. The photochemical quenching coefficient was calculated according to Schreiber et al.^44^ as qP = (F_m_'–F_t_)/(F_m_'–F_0_'). Stern–Volmer non-photochemical quenching was expressed as NPQ = (F_m_–F_m_')/F_m_'^45^.

The greenness index, which reflects the total chlorophyll content, was obtained using a portable chlorophyll meter (*Cl-01*, Hansatech) and determined using dual-wavelength optical absorbance (620 and 920 nm). The other technical parameters of the measurements are described by Borek et al.^46^. Regardless of the species, sampling was performed on the same leaves from the top parts of the plants that were used to measure chlorophyll a fluorescence. The sample sizes (number of biological replicates = plants) are listed in Table S7.

### Statistical analyses

Data were analyzed using the “lme4” library in R statistical software 4.1.1^47^ (Tables S8–S12).

To calculate the percentage of seed germination in both experiments (Experiment 1, Petri dishes and Experiment 2, flowerpots), we used a generalized linear mixed model (GLMM) with Gaussian error variance. The effects of the experimental treatment (control, goldenrod, walnut, and goldenrod and walnut), plant type (crop vs. weed), and the interaction term between the experimental treatment and plant type were included as fixed factors. Flowerpot identity (in Experiment 2) and plant species (in both experiments) nested in the plant type were included in the GLMM as random factors. Paired contrasts were used to find statistically significant differences between the levels of fixed factors using the package “lsmeans” in R.

Seed germination probability in Experiment 2 (greenhouse) was tested using a GLMM with binomial error variance. An identical model was used to test factors affecting the joint probability of seed germination and seedling survival. All dead seedlings were subtracted from germinated seeds and assigned a 0 in the analyses.

GLMMs with Gaussian error variance and identity links were used to test the effects of plant type, experimental treatment, and the interaction term between these factors on plant height and number of leaves. The random factors were the same as those used in the GLMM. A GLMM with Gaussian error variance and identity link was used to test the effect of plant type, experimental treatment, and the interaction term between these factors on seedling biomass, root system biomass, plant aboveground part biomass, relative biomass of the root system (biomass of root system divided by total seedling biomass), maximum efficiency of PS II–F_v_/F_m_, photochemical quenching (q_P_), non-photochemical quenching (NPQ), and greenness index. As only one seedling per pot was selected for these measurements, the only random factor was the plant species nested in the plant type. We also included the total seedling biomass as a covariate in the GLMM for the relative biomass of the root system.

### Plant collection statement

This study complied with institutional and Polish laws. No permission is required to collect parts of goldenrods, walnuts, or any other invasive plant species unless it is for the breeding of these species. Goldenrods and walnuts are not protected, and their parts were collected from abandoned fields in a region that is not under any protection by law.

Experiments were conducted using seeds of native plant species (weeds) and popular crops grown in Central Europe purchased by seed companies. We did not breed invasive species, nor did we use seeds or rhizomes. We did not use the seeds of the protected species or collect them from nature. All soil samples with invasive species extracts were disposed of at the university. We did not use any toxic chemicals in the experiments; therefore, our research did not fall under any laws in Poland or the EU.

## Results

Overall, we found that invasive plant extracts negatively affected the germination, growth, and photosynthesis of both weeds and crops.

### Walnut and goldenrod influence on germination, growth, and photosynthetic performance

**Figure 1:**
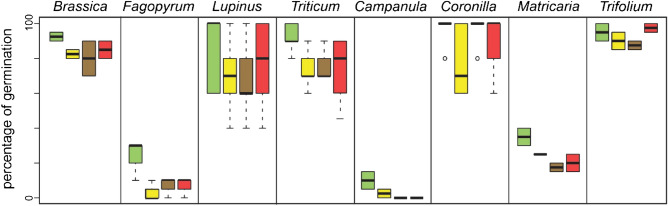
The percentage of seeds germinated for the species recorded in the Petri dish experiment. The four species on the left are crop species, while the four species on the right are weed species. The treatments are control (green), goldenrod extract (yellow), walnut extract (brown), and a combination of goldenrod and walnut extract (red). For all species, the same order of treatments is given, even if the box was too small to be colored. The boxes show the range of 50% of mean data with the horizontal line and the whiskers representing the median, and upper, and lower data limits without the outliers (circles), respectively.

We found that the extracts of both invasive species decreased seedling development, growth, and photosynthetic performance of the examined plant species (Table 1). The percentage of seed germination in Petri dishes with extracts from goldenrod and walnut leaves and their combination (in the following referred to as “extract treatments”) was lower than that in the control treatment (Fig. 1; Table S8a).

**Table 1:** Summary of effects of treatments in different experimental sets.

Effects	Goldenrod^1^	Walnut^1^	Mixed^1^	Crops/weeds^2^	Interaction between treatment and plant type
Percentage of germination P	▼	▼	▼as goldenrod	** = **	No
Probability of germination G	▼	▼**-** goldenrod	▼as goldenrod	** = **	Yes
Number of days needed for germination G	▲	▲	▲** + **goldenrod, as walnut	** = **	Yes
Probability of seedling death G	▲	▲	** = **	** = **	No
Probability of seedling survival for 2 weeks G	▼	▼**-** goldenrod	▼as goldenrod	** = **	No
Probability of seedling survival for 4 weeks G	▼	▼**-** goldenrod	▼as goldenrod	Weed species more affected than crop species	Yes
Height after 2 weeks G	▼	▼	▼as single treatments	** = **	Yes
Height after 4 weeks G	▼	▼	▼as single treatments	** = **	Yes
Number of leaves G	▼	▼	▼as single treatments	** = **	Yes
Leaf width after 2 weeks G	▼	▼	▼as single treatments	** = **	No
Leaf width after 4 weeks G ^3^	▼	▼	▼as single treatments	** = **	Yes
Total biomass G	▼	▼	▼as walnut	▼▲	Yes
Root biomass G	▼	▼	▼** + **single treatments	▼▲	Yes
Aboveground biomass G	▼	▼	▼as single treatments	▼▲	Yes
Proportion of root/total seedling biomass G	** = **	▼	** = **	▼▲	Yes
Maximum efficiency of PS II (Fv/Fm) G	▲	▲	▲ as single treatments	Stronger effects in crops than in weeds	No
Photochemical quenching (q_p_) G	▼▲	▼▲	▲** + **single treatments	** = **	No
Non-photochemical quenching (NPQ) G	▼▲	▼▲	▼▲ as single treatments	▼▲	No
Greenness index G	▲	▲	▲** + **single treatments	** = **	Yes

Differences between the control and all treatments with extracts (goldenrod, walnut, and the combination of goldenrod and walnut), as well as differences between the plant types (crops and weeds), are described. Interaction between treatment and plant type is reported. Symbols indicate an increase (▲), a decrease (▼), or a mixed reaction (▼▲); some are significantly positive and others significantly negative. Differences between the treatments are indicated as more than ( +) and less than (–) for the respective effect, while no effect ( =) refers to no differences between the control and the treatment or the crops vs. weeds groups. If the result was non-significant but showed a trend, the respective symbol is displayed in grey.

*P* Petri dish, *G* greenhouse flowerpots.

^1^Compared with control treatment (water).

^2^Comparison of weeds and crops subjected to any extracts of goldenrod, walnut, or both.

^3^Note that only three species, *Agrostemma*, *Fagopyrum*, and *Matricaria*, were studied for the second measurement of leaf width.

**Figure 2:**
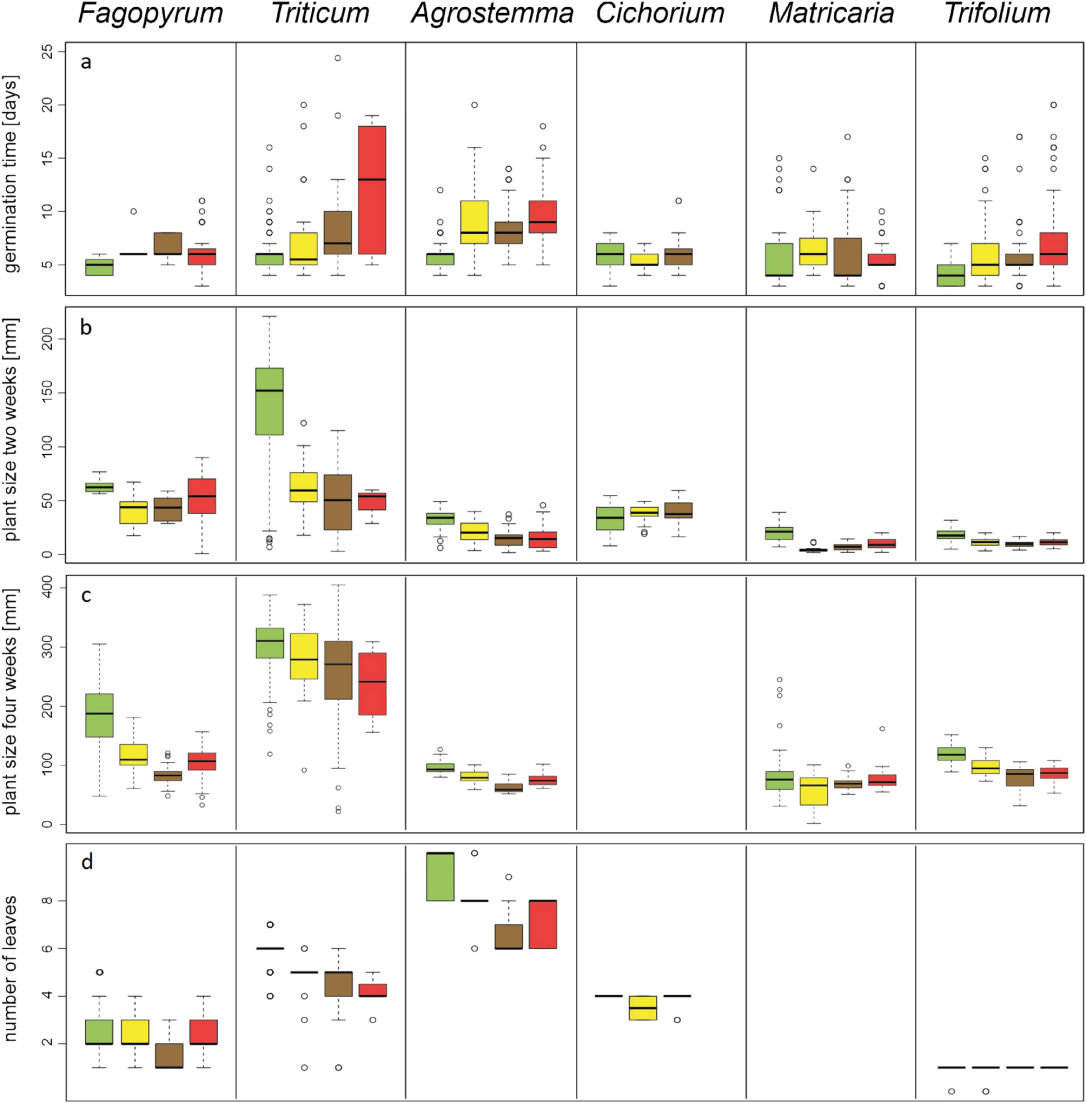
Germination time (**a**), plant size after two (**b**) and after 4 weeks (**c**) of experimental treatment, and the number of leaves (**d**) at the end of the experiment. The treatments are control (green), goldenrod extract (yellow), walnut extract (brown), and a combination of goldenrod and walnut extract (red). For all species, the same order of treatments is given, even if the box was too small to be colored. Note that no treatment with the combined extracts was available for *Cichorium* and that the plant size after 4 weeks was not measured for *Cichorium*, while the number of leaves was not determined for *Matricaria*. The boxes show the range of 50% of mean data with the horizontal line giving the median, and the whiskers representing the median, and upper, and lower data limits without the outliers (circles), respectively.

Seed germination probability (number of germinated sown seeds) also decreased with the extract treatments in the greenhouse experiment (Table S9). The probability of seedling death was higher in the pots treated with goldenrod extract than in the other treatments (Table S9). Seed germination in the greenhouse experiment was longer in the extract treatment than in the control treatment (Fig. 2a, Table S9).

**Figure 3:**
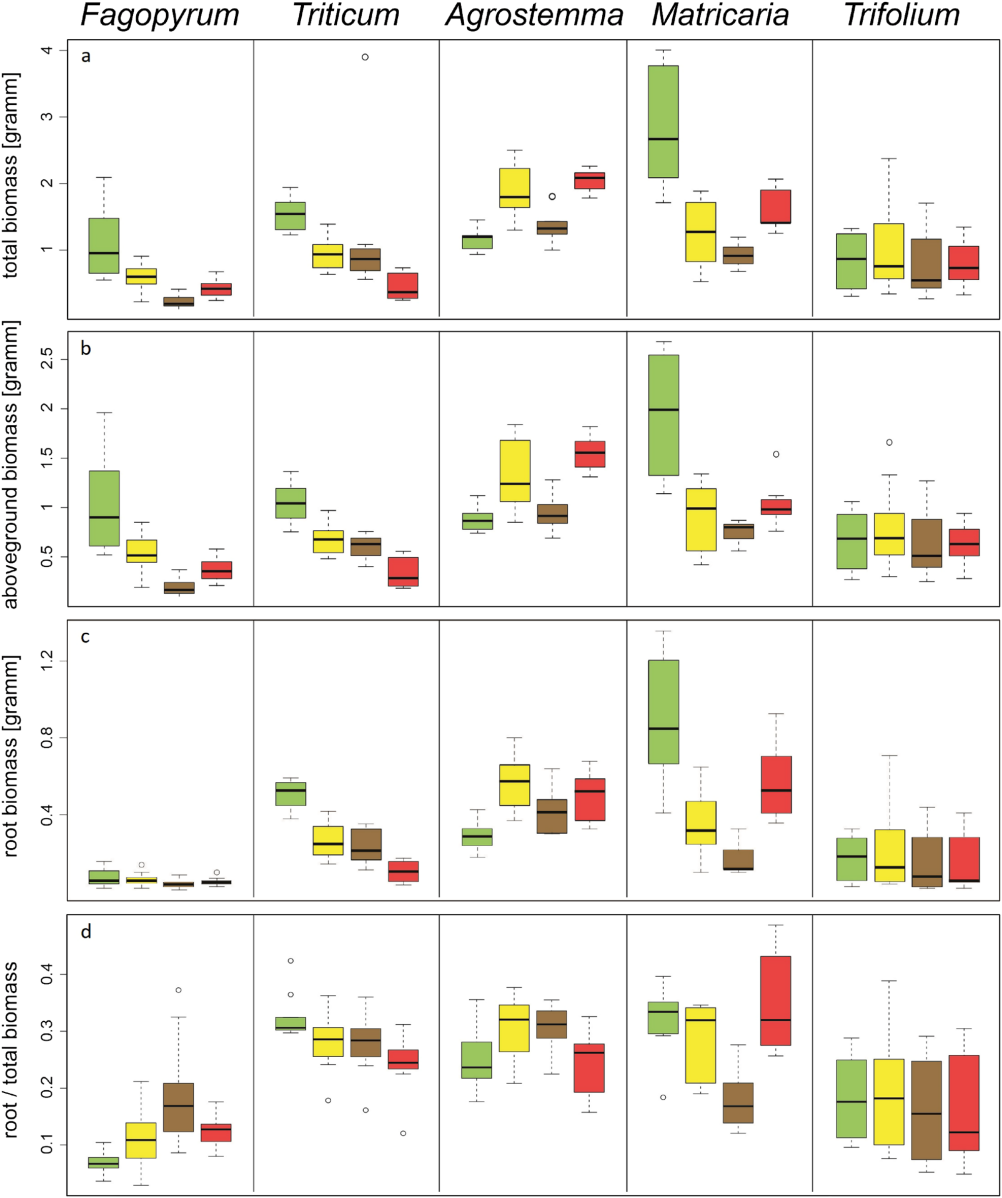
The measurements of biomass, i.e. total biomass (**a**), above-ground biomass (**b**), root biomass (**c**), and the relationship between root and total biomass (**d**). The treatments are control (green), goldenrod extract (yellow), walnut extract (brown), and a combination of goldenrod and walnut extract (red). For all species, the same order of treatments is given, even if the box was too small to be colored. The boxes show the range of 50% of mean data with the horizontal line giving the median, and the whiskers representing the median, and upper, and lower data limits without the outliers (circles), respectively.

Plants grown in the greenhouse with the extract treatments were shorter (Fig. 2b and c, Table S10) and had fewer leaves than those grown in the control treatment (Fig. 2d, Table S10). The total and aboveground biomasses of plants in the extract treatments were lower than those of the control (Fig. 3a and b, Table S10, Fig. S6). Root biomass was generally lower in the extract treatments, but higher in *Agrostemma*, resulting in only marginally significant overall results (Fig. 3c, Table S10). After controlling for total plant biomass, the relative proportion of the root system to total plant body biomass was significantly higher in the extract treatments than in the control for *Fagopyrum* but not for the other species (Fig. 3d, Table S10, Fig. S7).

**Figure 4:**
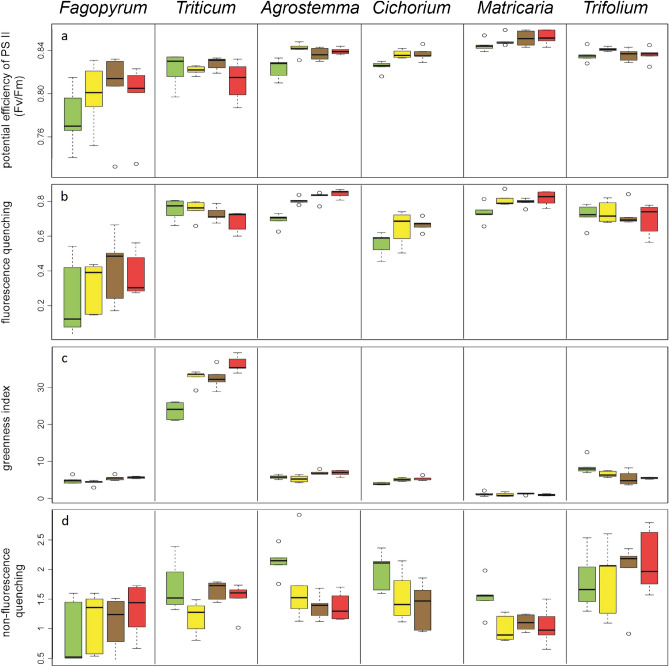
The measurements of photosynthesis-related factors, i.e. maximum efficiency of PS II (F_v_/F_m_) (**a**), photochemical quenching (q_P_) (**b**), greenness index (**c**), and non-photochemical quenching (NPQ) (**d**). The treatments are control (green), goldenrod extract (yellow), walnut extract (brown), and a combination of goldenrod and walnut extract (red). For all species, the same order of treatments is given, even if the box was too small to be colored. The boxes show the range of 50% of mean data with the horizontal line giving the median, and the whiskers representing the median, and upper, and lower data limits without the outliers (circles), respectively.

We found that the maximum efficiency of PS II (F_v_/F_m_) was lower in plants receiving the control treatment than in those receiving the extract treatments (Fig. 4a, Table S11). However, this effect was strongest for *Fagopyrum* and *Triticum* in the opposite direction, resulting in different responses according to the plant type (crop vs. weed; see below). Similar results were noted for photochemical quenching (Fig. 4b, Table S11) and greenness index (Fig. 4c, Table S11), whereas non-photochemical quenching showed no significant effect (Fig. 4d, Table S11). Only the greenness index was significantly influenced by the treatments and the interaction between plant type and treatment (Fig. 4c, Table S11).

### Are the goldenrod and walnut impacts on the germination, growth, and photosynthetic performance additive?

Our results suggest that the effects of the mixed extracts were similar to, or even less harmful than, the individual effects of either goldenrod or walnut (Table 1). In general, the mixed extracts of the invasive species decreased the percentage of germination in the Petri dish experiment, but the effect was not different from that of the single-species extracts (Table 1, Fig. 1, Tables S1, S5 and S9). Interestingly, *Trifolium repens* was not affected by the combined extracts, whereas it was affected by the single extract treatments (Fig. 1).

Similar effects were observed in the greenhouse experiments (Table 1, Fig. 2, and Table S10). Seed germination time was similar for the combined and single-species extract treatments, except for *Triticum*, in which germination time was prolonged for the combined treatment (Fig. 2a). The probability of seedling death in the mixed extracts was similar to that in plants grown in the control; however, both single-extract treatments showed a higher probability of seedling death. Overall, differences between the extract and control treatments were not statistically significant (Supplementary Table S9). When we included these dead seedlings in the calculation of the combined probability of germination and survival, we found that the probability was similar to that of the goldenrod treatment and lower than that of the walnut treatment (Tables 1 and S9). Plants in the greenhouse experiment for all extract treatments had similar heights (Table 1, Fig. 2a and b, Table S10), number of leaves (Table 1, Fig. 2d, Table S10), total seedling length, total seedling biomass, and aboveground and root biomass (Table 1, Fig. 3a–c, Table S11, Figs. S6, S7). Again, *Triticum* was an exception, as it showed reduced biomass in the combined treatment compared to the single extract treatments (Fig. 3a–c). For the maximum efficiency of PS II (Fv/Fm), photochemical quenching (q_P_), and greenness index, the combined extracts showed the strongest difference for *Triticum*; however, overall, the extract treatments were comparable (Tables 1, S11, Fig. 4a and b), and there was no difference in NPQ among the extract treatments (Fig. 4d).

### Crops versus weed details

Our data show that the extract treatment affected weeds and crop species similarly (Table 1). However, for long time surviving, weeds were more vulnerable than crop species. We found an increase in one of the photosynthetic measurements (F_v_/F_m_) in the weeds; however, the other three measurements showed no effect.

Accounting for the interaction between plant extract treatment and plant type (crop vs. weed), we identified different effects of the treatment on crop and weed plants for several parameters, such as the probability of seedling survival for 4 weeks, total biomass, root biomass, aboveground biomass, proportion of root/seedling biomass, maximum efficiency of PS II (F_v_/F_m_), and non-photochemical quenching (NPQ) (Table 1).

### Soil properties

During the experiment, the soil changed owing to the differences in the treatments (Table S12). For all treatments, including the control, the pH was less acidic, and the Mg content was higher at the end of the experiment. Phosphate was approximately one third higher in all invasive plant extract treatments than in the original soil but not in the control. The nitrogen content was lower in the control treatment than in the original soil and invasive plant treatments. Organic matter content increased when goldenrod extract was applied.

## Discussion

In the present study, we demonstrated the potential allelopathic effects of extracts from invasive plant species on the complementary and coexisting elements of a farmland ecosystem: crops and their accompanying floral weeds. In our novel approach, we used single-species extracts of invasive species, as well as a mixed extract of two species, to simulate environmental circumstances in both multi-invaded abandoned fields and extensively managed fields.

Decreased seed germination and stunted plant growth are two classic detrimental outcomes of allelopathy^29,32^. We found that extracts from invasive Canadian goldenrods and Persian walnuts lowered the probability of germination and decreased seedling growth of weeds and crops. Kocacë Aliskan and Terzi^48^ showed that seed germination of tomato (*Lycopersicon esculentum*), cucumber (*Cucumis sativus*), garden cress (*Lepidium sativum*)*,* and alfalfa (*Medicago sativa*) was strongly inhibited by walnut leaf extracts^48^. Interestingly, Terzi^49^ found that even decomposed walnut leaf extract inhibited the germination of cucumber seeds, which may be especially important for agriculture and the restoration of fields where walnuts grow. Juglone is responsible for allelopathy and is found in many plants of the walnut family *Juglandaceae,* including *J. nigra* and *J. regia*^34^.

Our finding that Canadian goldenrod leaf extract was harmful to seed germination and seedling growth is consistent with the results of Sun et al. who reported that germination of mulberry (*Morus alba*)*,* morning glory (*Pharbitis nil*), wheat (*Triticum aestivum*)*,* and field mustard (*Brassica campestris*) seeds were reduced by such extracts^35^. Early growth strategies can be critical for determining competitive interactions between species^50^, and thus, can be important in colonization and competition between invasive and native plants.

We also observed suppression of root growth caused by both goldenrod and walnut extracts. Kocacë Aliskan and Terzi^48^ showed similar effects as fresh walnut leaf extracts, and Terzi^49^ showed similar effects as decomposed walnut leaves. Invasive goldenrods also inhibit the root growth of lettuce *Lactuca sativa*^51,52^ and radish *Raphanus sativus*^51^. Slower development of the root system can decrease the ability of weeds and crops to reach deeper and moist soil layers. The phenomenon of a reduced root system in response to invasive species has already been observed in another invasive plant, the buckthorn, *Rhamnus cathartica,* on native herbs^53^.

Our results on photosynthetic performance are consistent with those of Cheng and Cheng^32^, who suggested that the effects of allelochemicals on plant photosynthesis mainly involve inhibition or damage to the biosynthesis machinery and acceleration of photosynthetic pigment decomposition. Allelochemicals affect photosynthesis, mainly by influencing PSII function^32^. In our study, the values of both the maximum efficiency of PS II (F_v_/F_m_) and photochemical fluorescence quenching (q_P_) were higher in crops and weeds growing on invasive species extracts than in the controls. Elevated levels of F_v_/F_m_ indicate that more PS II reaction centers are open and ready to utilize light energy^54^. The toxins in the extracts may be a signal of the occurrence of competitors that might switch photosynthesis to a high level, and a high rate of light conversion may be a terminal investment in the face of toxic neighbors. Elevated levels of NPQ noted in crops growing in walnut and mixed extracts suggest that plants balance the high chlorophyll content associated with high PSII activity with the possibility of using the obtained energy for photosynthesis via energy-suppression mechanisms within the photosynthetic apparatus^46^.

Only the goldenrod extract increased the probability of seedling death. Hypothetically, walnut allelopathic compounds may have fewer toxic effects; however, further research is needed to confirm this. Lenda et al.^4^ described the harmful effects of goldenrods and walnuts on the native plant biodiversity in abandoned fields. Only the goldenrod invasion caused a greater decrease in species richness and cover (74%) than the walnut invasion (58%)^4^.

In our study, mixed extracts did not affect the physiology or inhibit the growth of crops or weeds more than the single-species extracts. Moreover, they did not increase the seedling mortality rate. This might be explained by Lenda et al.^4^, who found that the combined impact of multi-invasion on native species diversity was much lower (15% decrease in native plant diversity) than 50% when goldenrod or walnut was used alone. Nevertheless, we found that the time taken for seeds to germinate following treatment with mixed extracts was the longest, which may be an important factor in shaping plant communities because fast germination is crucial for success in competition for resources and space. To the best of our knowledge, no other study has examined the effects of mixed extracts of co-invading plants on crops or weeds.

In the present study, the weeds appeared to be more resistant to extracts from the invasive species. This was especially apparent in characteristics such as the probability of seedling death, total biomass, relative allocation of root and aboveground biomass, and an increase in photosynthetic performance. The coevolution of invasive goldenrods and co-occurring weeds on abandoned land may explained this resistance in weeds. Invasive species may cause selective pressure, and only weeds that breed sexually and are not preselected by people can compete in this army race^55^. This indicates a stronger predisposition of weeds to competition. Therefore, crops may be more vulnerable to multiple invasions than weeds because weeds may compete better for water, soil, and light.

Allelopathy appears to be an important mechanism of plant invasion^27,56^; however, few invasive species coexist with crops. Callaway and Ridenour^27^ described the common allelopathic effect of invasive species on native species using their ‘novel weapons hypothesis,’ which suggests that allelochemicals from invasive plants have a negative effect on native plants because they have not yet evolved tolerance or resistance to these chemicals. Allelochemicals may directly inhibit the germination and growth of other plants^56^. Indirect effects have been noted when allelochemicals change interactions in the soil^56^, especially mycorrhizal associations^57^.

### Study limitations

There is an ongoing debate regarding allelopathy and how it should be studied in the field of ecology^32^. Some authors claim that allelopathic substances must be isolated and tested as individual chemicals^32^. However, other authors disagree because of the cumulative effect of plant tissues containing multiple chemicals that exhibit allelopathic effects^58^. The lack of consensus on how to study allelopathy and the lack of reliable methods are great challenges in enhancing our understanding of allelopathic effects on seeds and seedlings^58^. We followed the most commonly used methodology based on extracts. We know that there is no ideal method; however, using extracts closely mimics field conditions.

### Practical recommendations

Our study shows a strong allelopathic effect of invasive goldenrods and walnuts when they act separately; however, the allelopathic effect of their mixed extracts was neither additive nor synergistic. Such negative allelopathic effects of invasive species may be economically important because they affect crop plants. Invasive species that negatively affect native and wild flowering plants are a serious problem in nature conservation because such plants are important for the biodiversity of pollinators and birds, thereby improving the quality of ecosystem services in agricultural landscapes. Walnuts and goldenrods are allelopathic and should be managed in critical ecosystems. However, research has already shown that an invasion of goldenrod decreases the biodiversity of birds^59^, pollinators^14^, ants^60^, and plants^4^. We performed the study under experimental glasshouse conditions that may be treated as artificial compared to field studies to explore the additive effects of the two invasive species. Thus, further studies should explore the effects using field experiments. Nonetheless, our results suggest that action against the globally invasive goldenrod should be urgently planned and prioritized, especially in the European Union. Walnuts are invasive only in Central Europe and usually co-occur with goldenrods; thus, eradication efforts against the latter will be cost-efficient because they will also decrease wild-growing walnuts. Moreover, there is a need for research focusing on how long the allelopathic effect persists in the soil. This knowledge is important because invaded land requires specialized strategies for soil recultivation to minimize the toxic effects of these invasive species on crops and native flora in restored land. 

### Supplementary Information

Supplementary Information.

## Data Availability

Our data will be available in the Dryad Digital Repository (https://datadryad.org/) at the time of publication. Data analyzed will be made available on reasonable request to the corresponding author during the review process of this ms.
