# Supplementary material for: Multiple invasive species affect germination, growth, and photosynthesis of native weeds and crops in experiments
Source: Sci Rep. 2023 Dec 13;13:22146. doi: 10.1038/s41598-023-48421-w (PMC10719303; doi:10.1038/s41598-023-48421-w)
Supplement: Supplementary file 1 — Supplementary Information. [file 41598_2023_48421_MOESM1_ESM.docx]

**Supplementary Material**

**Detailed description of the methodology**

*1. Solidago canadensis and Juglans regia chemistry:*
Diterpenes are abundantly found in the members of *Solidago* genus and have been reported to have allelochemical activity (Harborne 1977, Hegnauer 1977, Herz, 1977). *S. canadensis* has been intensively studied for its chemical substances (Harborne 1977, Hegnauer 1977, Herz, 1977). *S. canadensis* L. contains solidagenone and a mixture of epimeric 9-13-oxygen-bridged dihydrofuranoid solidagenone derivatives. *S. canadensis* var. scabra (*S. altissima*) contains several trans-clerodane-type diterpenoids such as kolavenol, kolavenic acid, 6-oxokalavenic acid (altissimic acid), and the angelate of 6-hydroxykolavenic acid in its roots. The main bitter components of the roots, solidagnoic acid, is 7-acetoxykolavenic acid (Hegnauer 1977), and an aromadedrane derivative cyclocolorenon (Herz 1977).

*Juglans regia* (Juglandaceae; syn. common walnut, Persian walnut) originated from the area between the Black Sea Basin, Turkey, Central Asia, and the Himalayas, where it occurs in mixed and deciduous forests (Huntley & Birks 1983, Zohary & Hopf 1988). Walnut has attractive, tasty, fat seeds, and thus it has been cultivated for centuries outside its natural range, including Central Europe and North America. In Poland, walnuts were introduced in the Middle Ages to monasteries and grow well in Poland’s climatic and edaphic conditions (Carrion & Sanchez-Gomez 1992). There is extensive knowledge regarding juglone, an allelopathic substance produced by walnut. Juglone causes biodiversity declines because it inhibits the growth of many other species (Vander Wall 2001, Ercisli et al. 2005). All parts of this plant, including leaves and flowers, contain juglone (Ponder & Tadros 1985, Ercisli et al. 2005). The walnut has pinnate foliage, is wind-pollinated, and its male flowers are catkins. Its pollen frequently causes serious allergic effects (Pastorello et al. 2004). Walnut is sensitive to low temperatures and can be damaged by frost late in spring (e.g. in May) when the leaves and flowers emerge. The tree starts bearing seeds when it is 8-10 years old and the seeds, popularly called nuts, ripen during September and October (Huntley & Birks 1983; Zohary & Hopf 1988; Carrion & Sanchez-Gomez 1992).
*2. Measuring plant photosynthetic performance*

Chlorophyll a fluorescence parameters and chlorophyll content were estimated photometrically as a greenness index. We used the following characteristics: 1) potential efficiency of PS II (F_v_/F_m_), 2) fluorescence quenching (q_P_), 3) non-fluorescence quenching (NPQ), and 4) greenness index. Chlorophyll fluorescence parameters were measured using an *FMSII* pulse-amplitude modulated fibre-optic system (*Hansatech*, Kings Lynn, UK). Leaf clips (with a 5-mm diameter hole) were fastened on leaves and kept there for 20 min for dark adaptation. Minimal fluorescence in the dark-adapted state (F_0_) was measured, and a saturating-light pulse (10,000 μmol(photon) m^−2^ s^−1^ for 0.9 s) was used to determine maximal fluorescence in the dark-adapted state (F_m_). Next, the leaf was irradiated with actinic light [1,500 μmol(photon) m^−2^ s^−1^] for 270 s to measure its steady-state fluorescence (F_t_). Then, the minimal fluorescence yield in the light-adapted state (F_0_') was measured by immediately irradiating the leaf for 3 s with a far-red emitting diode (radiation of about 15 W m^–2^). Next, the saturating-light pulse was used again to determine the maximal fluorescence yield in the light-adapted state (F_m_’).

The maximum efficiency of PSII photochemistry was calculated as F_v_/F_m_, where F_v_ = F_m_ – F_0_.

The photochemical quenching coefficient was calculated according to Schreiber et al. (1986): q_P_ = (F_m_' – F_t_)/(F_m_' – F_0_'). Stern-Volmer non-photochemical quenching was expressed as NPQ = (F_m_ – F_m_')/F_m_' (Bilger and Björkman 1991). The greenness index was obtained with a portable chlorophyll meter (*Cl-01*, *Hansatech,* UK), and determined using dual-wavelength optical absorbance (620 and 920 nm). Other technical parameters of the measurements are described in Borek et al. 2016. All measurements were performed on the same leaves from the top part of the plants regardless of the species. Sample sizes are given in Table S7.

**Statistical analysis**

To calculate the percentage of seed germination in both experiments (experiment 1, Petri dishes and experiment 2, flowerpots) we used a generalised linear mixed model (GLMM) with gaussian error variance. The effects of experimental treatment (levels: control, goldenrod, walnut, and goldenrod and walnut), plant type (crop vs weed), and interaction term between experimental treatment and plant type were included as fixed factors. Flowerpot identity (in experiment 2) and plant species (for both experiments) nested in plant type were included in GLMM as random factors. Paired contrasts were used to find statistically significant differences between the levels of fixed factors using the package “lsmeans” in R.

Seed germination probability in experiment 2 (green house) was tested using a generalised linear mixed model (GLMM) with binomial error variance.

An identical model was used to test factors affecting the joint probability of seed germination and seedling survival. In this case, all seedlings that died were subtracted from germinated seeds and assigned a 0 in the analyses.

GLMMs with Gaussian error variance and identity link were used to test the effect of plant type, experimental treatment, and interaction term between these factors on plant height and number of leaves. The random factors were the same as those in the GLMM above. GLMM with Gaussian error variance and identity link was used to test the effect of plant type, experimental treatment, and interaction term between these factors on seedling weight, root system weight, plant aboveground part weight, relative weight of root system (mass of root system divided by total seedling mass), standardised potential efficiency of PS II – Fv/Fm, standardised fluorescence quenching (q_P_), standardised non-fluorescence quenching (NPQ), and standardised greenness index. Since only one seedling per pot was selected for these measurements, the only random factor was plant species nested in plant type. In GLMM for the relative weight of the root system, we also included total seedling weight as a covariate.

GLMMs were done and visualised in R software (R Core Team 2017).

**Supplementary Tables and Figures**

Table S1. Sample size (number of seeds sown) for estimating seed germination probability following different treatments performed in Petri dishes

| Species\| Treatment | Control | Goldenrod | Walnut | Goldenrod and walnut | **Total** |
| --- | --- | --- | --- | --- | --- |
| *Brassica oleracea* (C) | 40 | 40 | 40 | 40 | 160 |
| *Campanula patula* (W) | 40 | 40 | 40 | 40 | 160 |
| *Coronilla varia* (W) | 30 | 30 | 30 | 30 | 120 |
| *Fagopyrum esculentum* (C) | 30 | 30 | 30 | 30 | 120 |
| *Lupinus album* (C) | 30 | 30 | 30 | 30 | 120 |
| *Matricaria chamomilla* (W) | 40 | 40 | 40 | 40 | 160 |
| *Trifolium repens* (W) | 40 | 40 | 40 | 40 | 160 |
| *Triticum sp.* (C) | 50 | 50 | 50 | 50 | 200 |
| **Total** | 300 | 300 | 300 | 300 | 1,200 |

The letter in brackets indicates whether the species is a weed (W) or a crop (C).

Table S2. Sample size (number of seeds sown) in the green house experiment to estimate seed germination percentage, seedling death probability, and joint seed germination as well as survival and morphological data following different treatments.

| Species\| Treatment | Control | Goldenrod | Walnut | Goldenrod and walnut | **Total** |
| --- | --- | --- | --- | --- | --- |
| *Agrostemma githago* (W) | 180 | 180 | 180 | 180 | 720 |
| *Cichorium intybus* (W) | 34 | 34 | 34 | 0 | 102 |
| *Fagopyrum esculentum* (C) | 181 | 180 | 180 | 183 | 724 |
| *Lupinus album* (C) | 180 | 180 | 180 | 180 | 720 |
| *Matricaria chamomilla* (W) | 180 | 180 | 180 | 180 | 720 |
| *Trifolium repens* (W) | 186 | 183 | 180 | 180 | 729 |
| *Triticum sp.* (C) | 180 | 180 | 181 | 180 | 721 |
| **Total** | 1,121 | 1,117 | 1,115 | 1,083 | 4,436 |

The letter in brackets indicates whether the species is a weed (W) or a crop (C).

Table S3. Sample size (number of plants examined) to estimate the number of days required for seeds to germinate (germination time) following different treatments. The given values in brackets are the percentages of germinated plants. Note that *Lupinus* was excluded in the model calculation as in the control treatment germination was reduced due to a technical issue.

| Species\| Treatment | Control | Goldenrod | Walnut | Goldenrod and walnut | **Total** |
| --- | --- | --- | --- | --- | --- |
| *Agrostemma githago* (W) | 172 (95.6%) | 112 (62.2%) | 128 (71.1%) | 131 (72.8%) | 543 (75.4%) |
| *Cichorium intybus* (W) | 7 (20.6%) | 6 (17.6%) | 10 (29.4%) | 0 | 23 (22.5%) |
| *Fagopyrum esculentum* (C) | 85 (47.0%) | 24 (13.3%) | 33 (18.3%) | 35 (19.1%) | 177 (24.4%) |
| *Lupinus album* (C) | 4 (2.2%) | 16 (8.9%) | 41 (22.8%) | 13 (7.2%) | 74 (10.3%) |
| *Matricaria chamomilla* (W) | 34 (18.9%) | 16 (8.9%) | 23 (12.8%) | 27 (15%) | 100 (13.9%) |
| *Trifolium repens* (W) | 145 (78.0%) | 80 (43.7%) | 103 (57.2%) | 92 (51.1%) | 420 (57.6%) |
| *Triticum sp.* (C) | 124 (68.9%) | 30 (16.7%) | 88 (48.6%) | 8 (4.4%) | 250 (34.7%) |
| **Total** | 571 (50.9%) | 284 (25.4%) | 426 (38.2%) | 306 (28.3%) | 1,587 (35.77%) |

The letter in brackets after the species names indicates whether the species is a weed (W) or a crop (C).

Table S4. Sample size (number of plants examined) to estimate the height of seedlings following different treatments. Note that *Lupinus* was excluded in the model calculation as in the control treatment germination was reduced due to a technical issue.

| Species\| Treatment | Control | Goldenrod | Walnut | Goldenrod and walnut | **Total** |
| --- | --- | --- | --- | --- | --- |
| *Agrostemma githago* (W) | 169 | 63 | 80 | 49 | 361 |
| *Cichorium intybus* (W) | 7 | 6 | 10 | 0 | 23 |
| *Fagopyrum esculentum* (C) | 77 | 24 | 33 | 33 | 167 |
| *Lupinus album* (C) | 2 | 10 | 37 | 9 | 58 |
| *Matricaria chamomilla* (W) | 23 | 13 | 18 | 22 | 76 |
| *Trifolium repens* (W) | 142 | 73 | 98 | 80 | 393 |
| *Triticum sp.* (C) | 117 | 26 | 62 | 3 | 208 |
| **Total** | 537 | 215 | 338 | 196 | 1,286 |

The letter in brackets indicates whether the species is a weed (W) or a crop (C).

Table S5. Sample size (number of plants examined) to estimate the number of leaves on seedlings following different treatments. Note that *Lupinus* was excluded in the model calculation as in the control treatment germination was reduced due to a technical issue.

| Species\| Treatment | Control | Goldenrod | Walnut | Goldenrod and walnut | **Total** |
| --- | --- | --- | --- | --- | --- |
| *Agrostemma githago* (W) | 15 | 15 | 15 | 15 | 60 |
| *Cichorium intybus* (W) | 7 | 6 | 10 | 0 | 23 |
| *Fagopyrum esculentum* (C) | 77 | 24 | 30 | 33 | 164 |
| *Lupinus album* (C) | 2 | 10 | 37 | 9 | 58 |
| *Trifolium repens* (W) | 142 | 73 | 98 | 80 | 393 |
| *Triticum sp.* (C) | 120 | 25 | 77 | 8 | 230 |
| **Total** | 363 | 153 | 267 | 145 | 928 |

The letter in brackets indicates whether the species is a weed (W) or a crop (C).

Table S6. Sample size (number of plants examined) for measuring plant biomass of the root system and aboveground parts following different treatments. Note that *Lupinus* was excluded in the model calculation as in the control treatment germination was reduced due to a technical issue.

| Species\| Treatment | Control | Goldenrod | Walnut | Goldenrod and walnut | **Total** |
| --- | --- | --- | --- | --- | --- |
| *Agrostemma githago* (W) | 10 | 10 | 10 | 10 | 40 |
| *Fagopyrum esculentum* (C) | 20 | 16 | 16 | 16 | 68 |
| *Lupinus album* (C) | 2 | 9 | 16 | 9 | 36 |
| *Matricaria chamomilla* (W) | 8 | 7 | 7 | 7 | 29 |
| *Trifolium repens* (W) | 10 | 11 | 11 | 11 | 43 |
| *Triticum sp.* (C) | 10 | 14 | 11 | 8 | 43 |
| **Total** | 60 | 67 | 71 | 61 | 259 |

The letter in brackets indicates whether the species is a weed (W) or a crop (C).

Table S7. Sample size (number of plants examined) for measuring plant metabolism following different treatments

| Species\| Treatment | Control | Goldenrod | Walnut | Goldenrod and walnut | **Total** |
| --- | --- | --- | --- | --- | --- |
| *Agrostemma githago* (W) | 5 | 5 | 5 | 5 | 20 |
| *Cichorium intybus* (W) | 5 | 4 | 5 | 0 | 14 |
| *Fagopyrum esculentum* (C) | 5 | 5 | 5 | 5 | 20 |
| *Lupinus album* (C) | 3 | 4 | 5 | 5 | 17 |
| *Matricaria chamomilla* (W) | 5 | 5 | 5 | 6 | 21 |
| *Trifolium repens* (W) | 5 | 5 | 5 | 5 | 20 |
| *Triticum sp.* (C) | 5 | 5 | 5 | 5 | 20 |
| **Total** | 33 | 33 | 35 | 31 | 132 |

The letter in brackets indicates whether the species is a weed (W) or a crop (C).

Table S8. The percentage of germination in experiment 1 (Petri dishes) using generalized linear mixed effects model with species identity as random factor.

| Source of variation | F | df1, df2 | P |
| --- | --- | --- | --- |
| Plant type | 0.113 | 1, 6 | 0.75 |
| Treatment | 6.374 | 3, 98 | <0.001 |
| Plant type × treatment | 0.875 | 3, 98 | 0.457 |

Results from the generalised linear mixed effects model F: F test statistics

df, degree of freedom; P, statistical significance of the test

Table S8a Multiple comparison of the effects of treatment in the petri dish experiment (model presented in Table S8).

| Contrast | Estimate | SE | df | t-value | P |
| --- | --- | --- | --- | --- | --- |
| control~goldenrod | 15.31 | 3.67 | 98 | 4.178 | 0.0004 |
| control~walnut | 11.51 | 3.67 | 98 | 3.140 | 0.0118 |
| control~goldenrod + walnut | 10.26 | 3.67 | 98 | 2.799 | 0.0309 |
| goldenrod~walnut | -3.8 | 3.67 | 98 | -1.037 | 0.728 |
| goldenrod~goldenrod + walnut | -5.05 | 3.67 | 98 | -1.378 | 0.5159 |
| walnut~goldenrod + walnut | -1.25 | 3.67 | 98 | -0.341 | 0.9863 |

Table S9. The effect of plant type (crop or weed), experimental treatment, and interaction term on vital parameters of seeds and seedlings of studied plant species. Note that *Lupinus albus* was excluded in the model calculations as in the control treatment germination was reduced due to a technical issue.

| *Probability of germination* |  |  |  |
| --- | --- | --- | --- |
| Source of variation | sum of squares | Mean of squares | F |
| Plant type | 0.29 | 0.29 | 0.29 |
| Treatment | 239.4 | 79.8 | 79.8 |
| Plant type × treatment | 35.5 | 11.8 | 11.8 |
| *Number of days required for seeds to germinate* |  |  |  |
|  | F | df1, df2 | P |
| Plant type | 0.014 | 1, 4 | 0.91 |
| Treatment | 39.9 | 3, 821 | <0.001 |
| Plant type × treatment | 4.49 | 3, 821 | 0.004 |
| *Probability of seedling death* |  |  |  |
|  | sum of squares | Mean of squares | F |
| Plant type | 0.71 | 0.71 | 0.71 |
| Treatment | 11.21 | 3.74 | 3.74 |
| Plant type × treatment | 1.13 | 0.38 | 0.38 |
| *Probability that seeds germinate and survive after two weeks (first measurement)* |  |  |  |
| Plant type | 0.14 | 0.14 | 0.14 |
| Treatment | 313.4 | 104.5 | 104.5 |
| Plant type × treatment | 8.41 | 2.80 | 2.80 |
| *Probability that seeds germinate and survive after four weeks (second measurement)* |  |  |  |
| Plant type | 6.67 | 6.67 | 6.67 |
| Treatment | 93.5 | 31.2 | 31.2 |
| Plant type × treatment | 76.1 | 25.4 | 25.4 |
| *Height of seedlings (first measurement)* |  |  |  |
|  | F | df1, df2 | P |
| Plant type | 3.34 | 1, 4 | 0.14 |
| Treatment | 177.1 | 1, 807 | <0.001 |
| Plant type × treatment | 72.4 | 1, 807 | <0.001 |
| *Height of seedlings (second measurement)* |  |  |  |
| Plant type | 4.7 | 1, 3 | 0.12 |
| Treatment | 45.3 | 3, 644 | <0.001 |
| Plant type × treatment | 6.50 | 3, 644 | <0.001 |
| *Number of leaves on seedlings* |  |  |  |
| Plant type | 0.004 | 1, 4 | 0.96 |
| Treatment | 50.9 | 3, 334 | <0.001 |
| Plant type × treatment | 16.4 | 3, 334 | <0.001 |
| *Leaf width (first measurement)* |  |  |  |
| Plant type | 0.72 | 1, 3 | 0.46 |
| Treatment | 30.42 | 3, 488 | <0.001 |
| Plant type × treatment | 1.74 | 3, 488 | 0.16 |
| *Leaf width (second measurement)* |  |  |  |
| Plant type | 0.21 | 1, 1 | 0.72 |
| Treatment | 3.83 | 3, 191 | 0.011 |
| Plant type × treatment | 5.63 | 3, 191 | 0.0011 |

The results are from general linear mixed models. In cases with presence/absence data a model with binominal error distribution was used. Experiment were conducted using flowerpots set in a glasshouse. For further explanation, see Table S8.

Table S10. The effects of plant type (crop or weed), experimental treatment, and interaction term on total plant mass, root mass, aboveground plant height, and relative size (proportion) of the root system in total plant mass for those plants removed at week 4 (after second measurement, i.e., a subset of the plants measured above).

| *Plant height* |  |  |  |
| --- | --- | --- | --- |
| Source of variation | F | df1, df2 | P |
| Plant type | 1.58 | 1, 3 | 0.30 |
| Treatment | 53.7 | 3, 212 | <0.001 |
| Plant type × treatment | 13.1 | 3, 212 | <0.001 |
| *Total plant mass* |  |  |  |
| Plant type | 2.91 | 1, 3 | 0.19 |
| Treatment | 17.7 | 3, 212 | <0.001 |
| Plant type × treatment | 6.27 | 3, 212 | <0.001 |
| *Root mass* |  |  |  |
| Plant type | 1.91 | 1, 3 | 0.26 |
| Treatment | 6.12 | 3, 212 | <0.001 |
| Plant type × treatment | 2.37 | 3, 212 | 0.071 |
| *Aboveground plant mass* |  |  |  |
| Plant type | 3.58 | 1, 3 | 0.16 |
| Treatment | 21.1 | 3, 212 | <0.001 |
| Plant type × treatment | 8.57 | 3, 212 | <0.001 |
| *Proportion of root mass* |  |  |  |
| Plant type | 0.33 | 1, 3 | 0.61 |
| Treatment | 0.74 | 3, 212 | 0.53 |
| Plant type × treatment | 4.08 | 3, 212 | 0.008 |

Experiments were conducted using flowerpots in a glasshouse. Results from general linear mixed models. For other explanations, see Table S8.

Table S11. The effects of plant type (crop or weed), experimental treatment, and interaction term on potential efficiency of PS II photosystem (F_v_/F_m_), fluorescence quenching (q_P_), non-fluorescence quenching (NPQ), and greenness index

| *Fv/Fm* |  |  |  |
| --- | --- | --- | --- |
| Source of variation | sum of squares | Mean of squares | F |
| Plant type | 0.0025 | 0.0025 | 9.94 |
| Treatment | 0.0007 | 0.0020 | 2.70 |
| Plant type × treatment | 0.0002 | 0.0005 | 0.62 |
| *q_P_* |  |  |  |
| Plant type | 0.02 | 0.02 | 2.26 |
| Treatment | 0.03 | 0.10 | 3.96 |
| Plant type × treatment | 0.003 | 0.010 | 0.40 |
| *NPQ* |  |  |  |
| Plant type | 0.20 | 0.20 | 1.07 |
| Treatment | 0.49 | 1.46 | 2.61 |
| Plant type × treatment | 0.32 | 0.97 | 1.75 |
| *Greenness index* |  |  |  |
| Plant type | 10.4 | 10.4 | 2.66 |
| Treatment | 22.7 | 68.0 | 5.79 |
| Plant type × treatment | 57.1 | 171.3 | 14.59 |

Results from general linear mixed models. For other explanations, see Table S8.


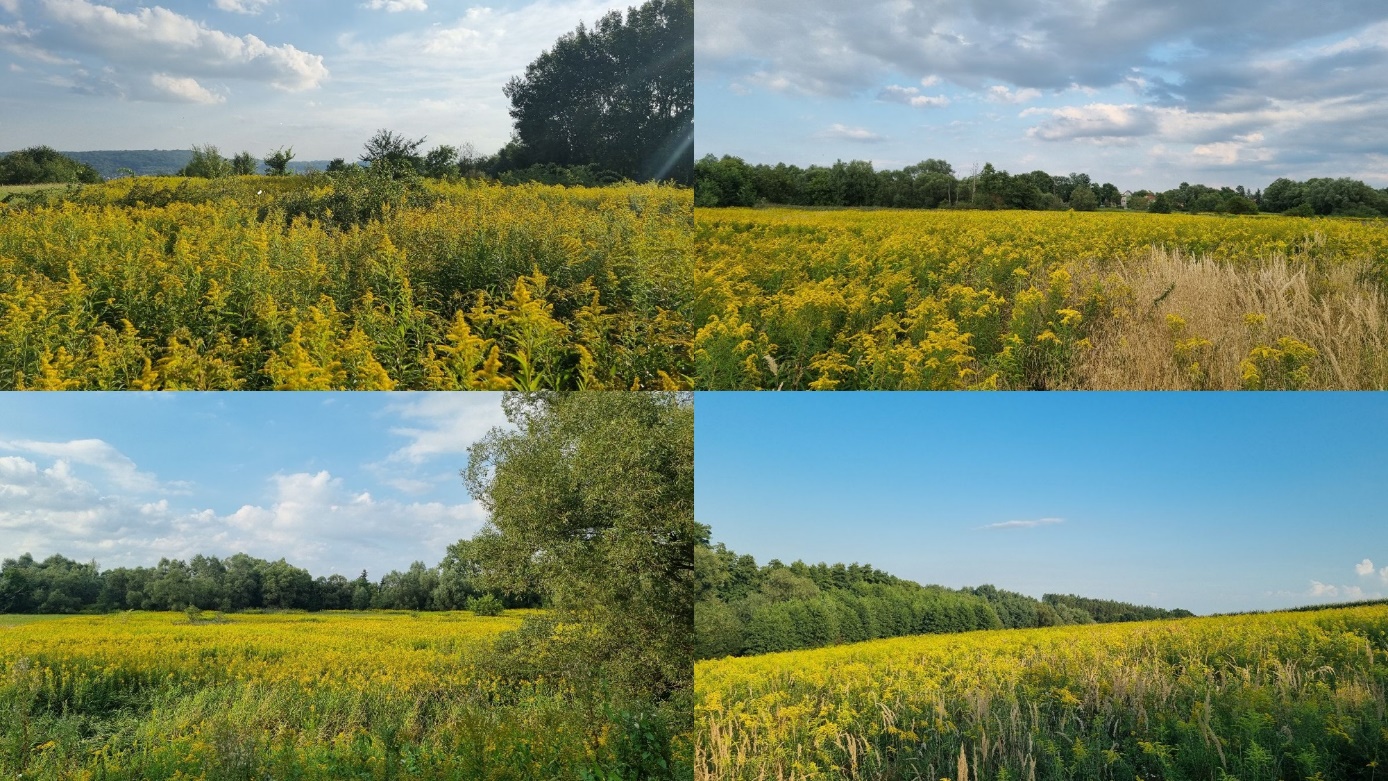


Fig. S1. Examples of goldenrod forming large areas of monoculture at abandoned land areas in Poland. All photos by Piotr Skórka


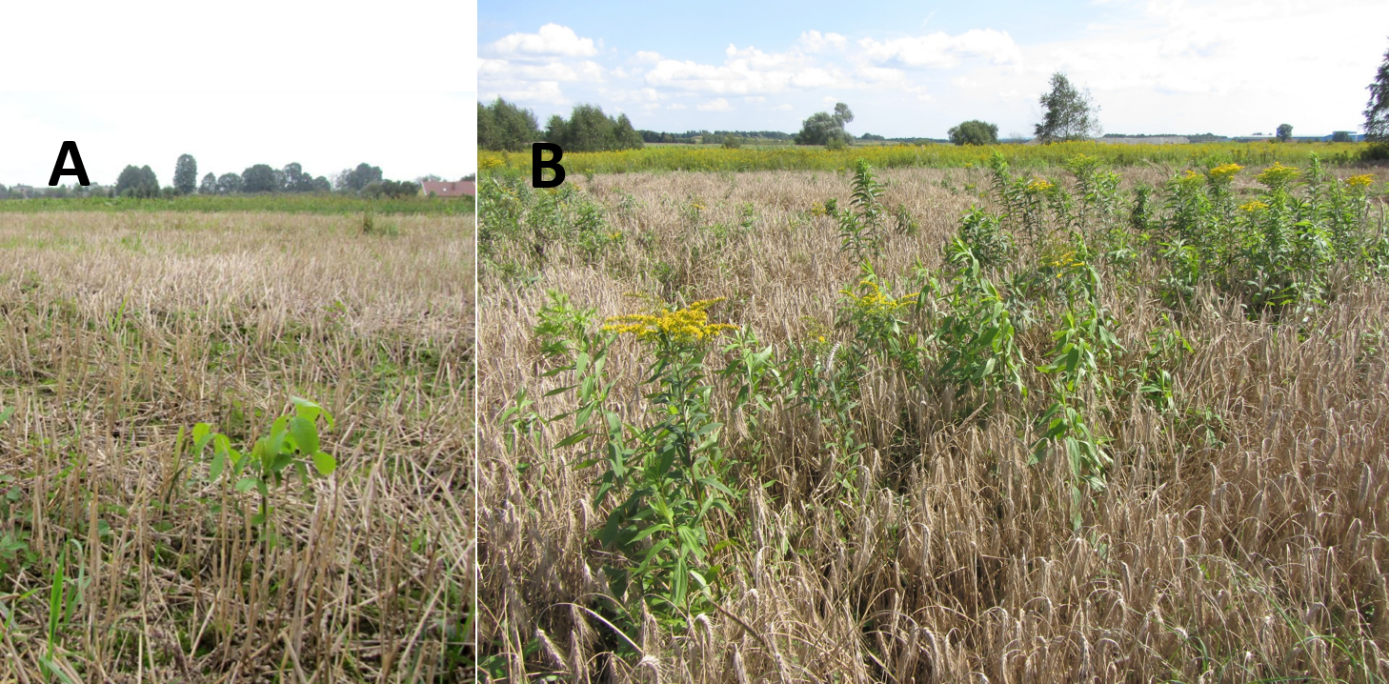

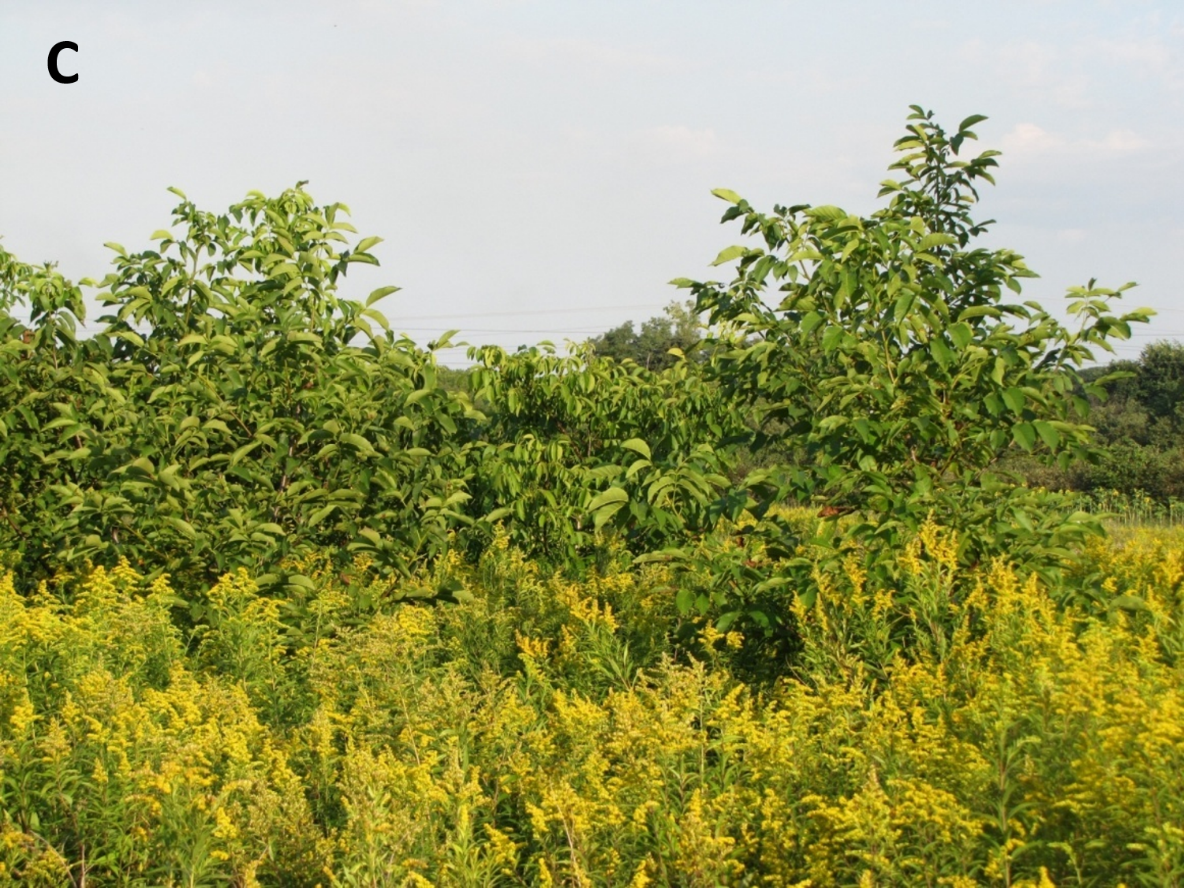


Fig S2. Walnut (A) and goldenrod (B) growing in managed agricultural fields. Walnut and goldenrod multi-invade abandoned agricultural fields (C). All photos by Piotr Skórka
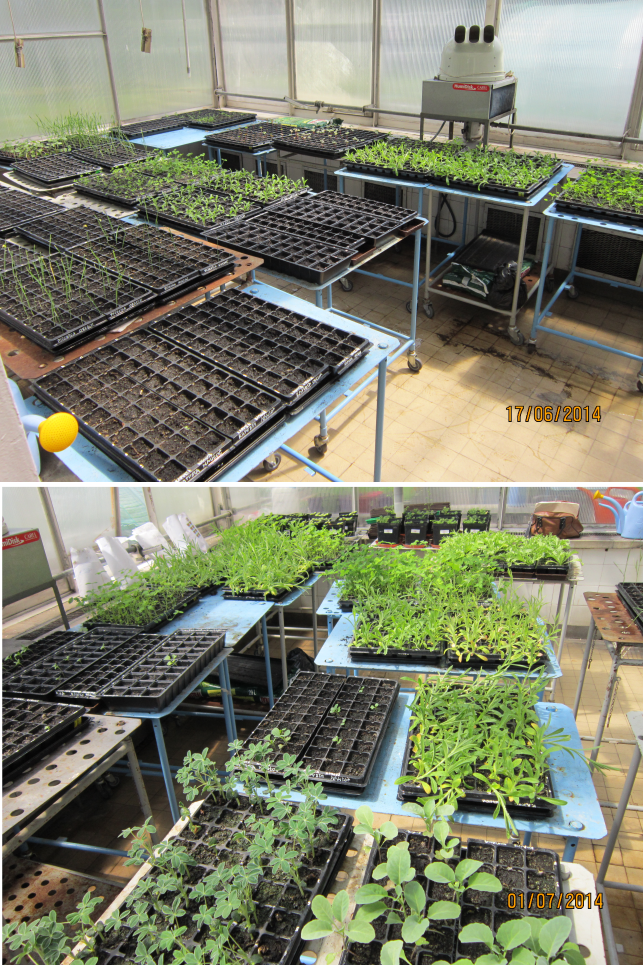

 Figure S3. Part of the greenhouse with flowerpots. Photos by Zuzanna Zagrodzka.


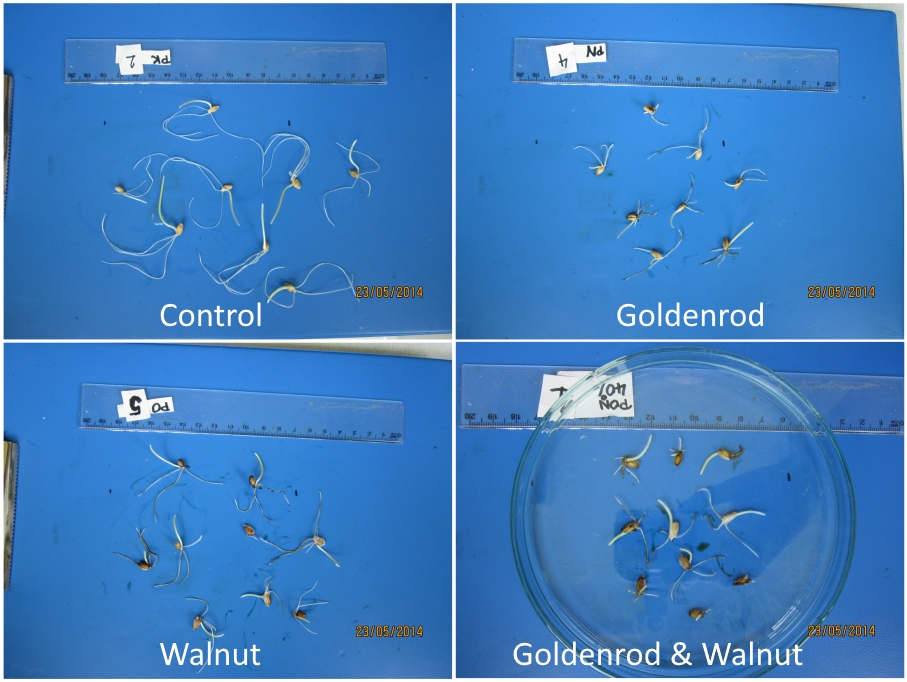


Figure S4. The effect of treatment on the development of *Triticum sp.* seeds sown in Petri dishes. Treatments included water (control), extracts from goldenrod, extracts from walnut, and mixed extracts from both species. Note the different magnification of the goldenrod and walnut treatment. Photos by Zuzanna Zagrodzka.


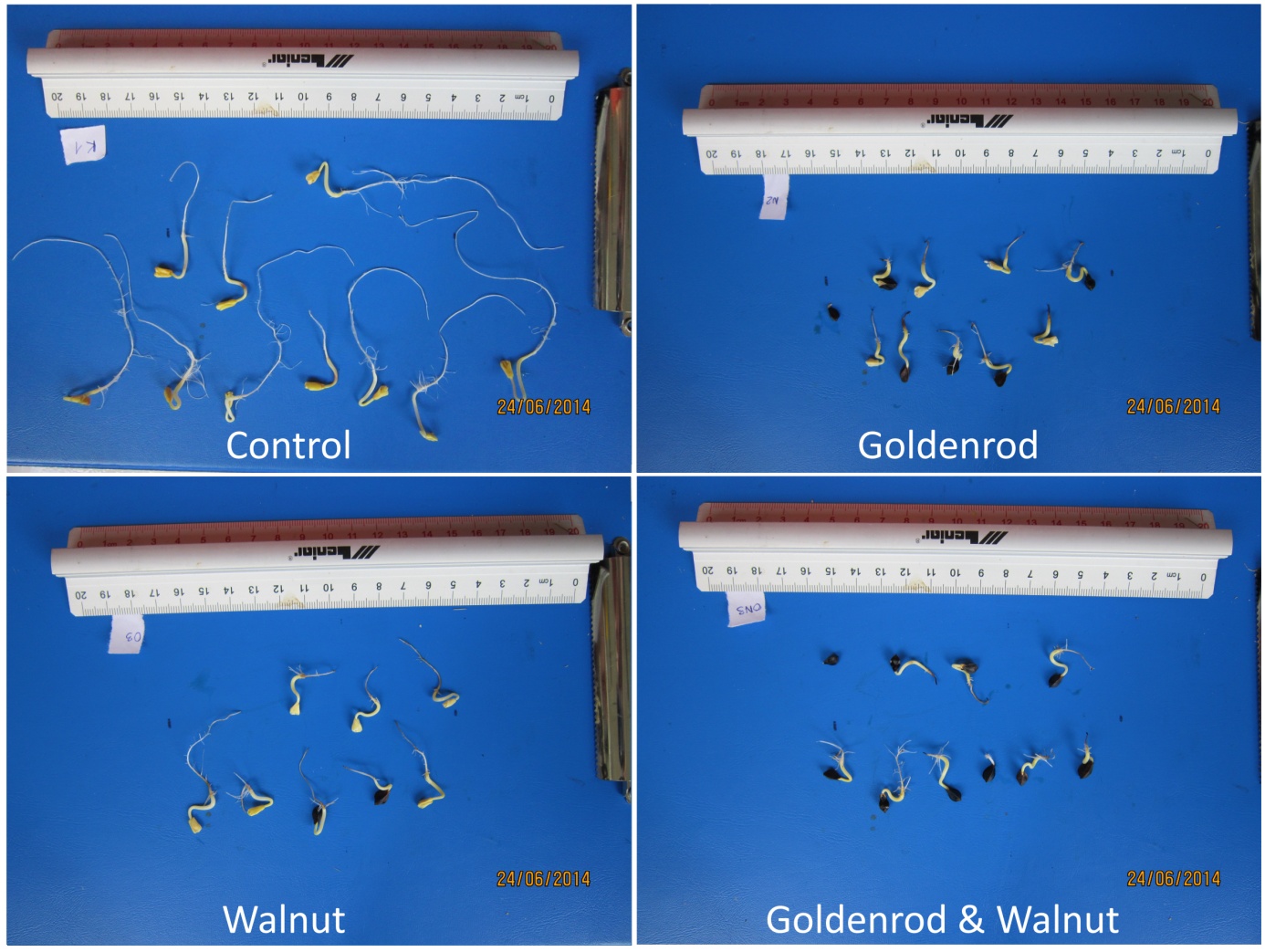


Figure S5. The effect of treatment on the development of *Fagopyrum esculentum* seeds growing in Petri dishes. Treatments were water (control), extracts from goldenrod, extracts from walnut, and mixed extracts from both the species. Photos by Zuzanna Zagrodzka.


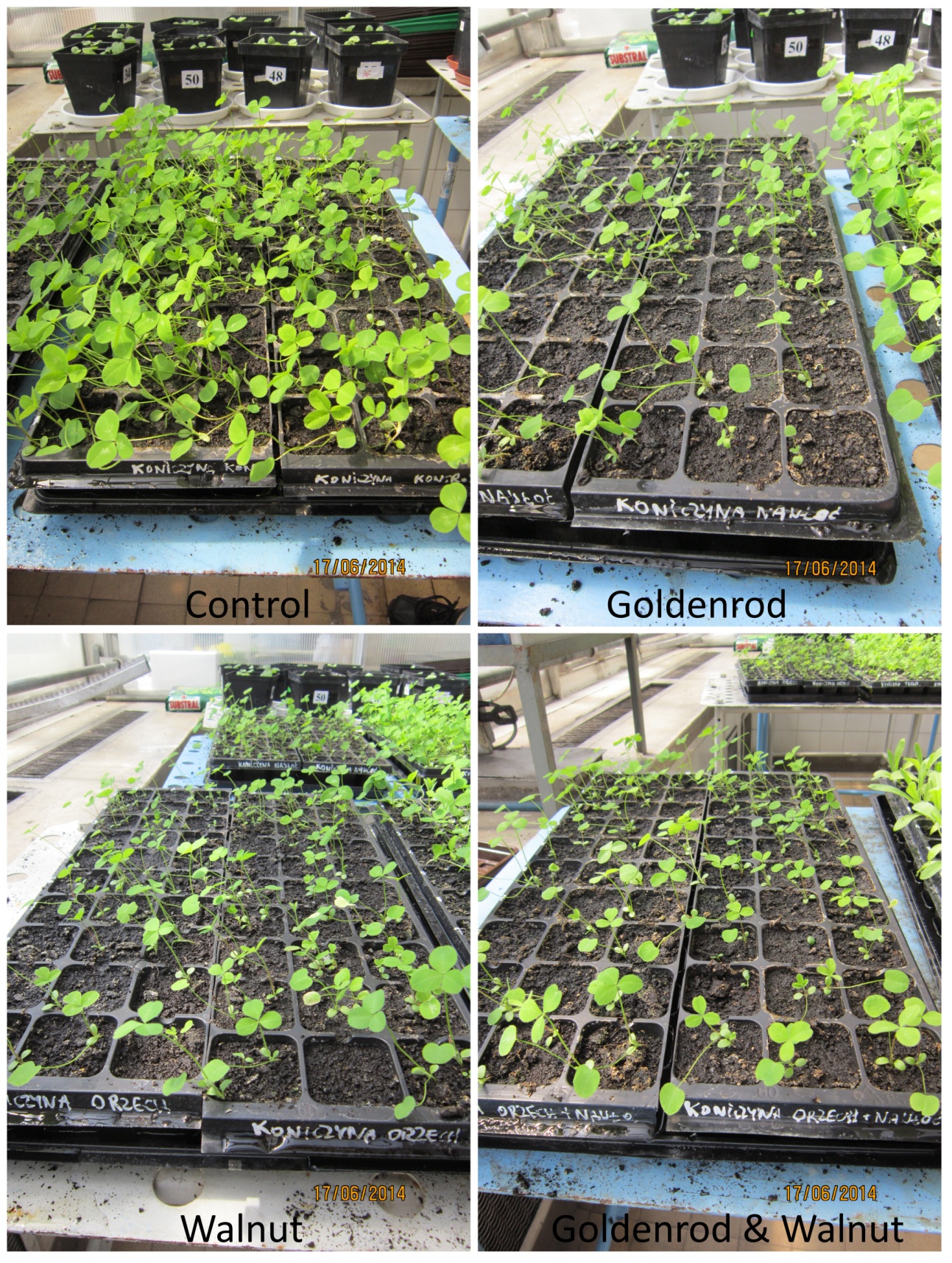


Figure S6. The effect of treatment on the development of *Trifolium repens* seeds growing in flowerpots. Treatments were water (control), extracts from goldenrod, extracts from walnut, and mixed extracts from the both species. Photos by Zuzanna Zagrodzka.


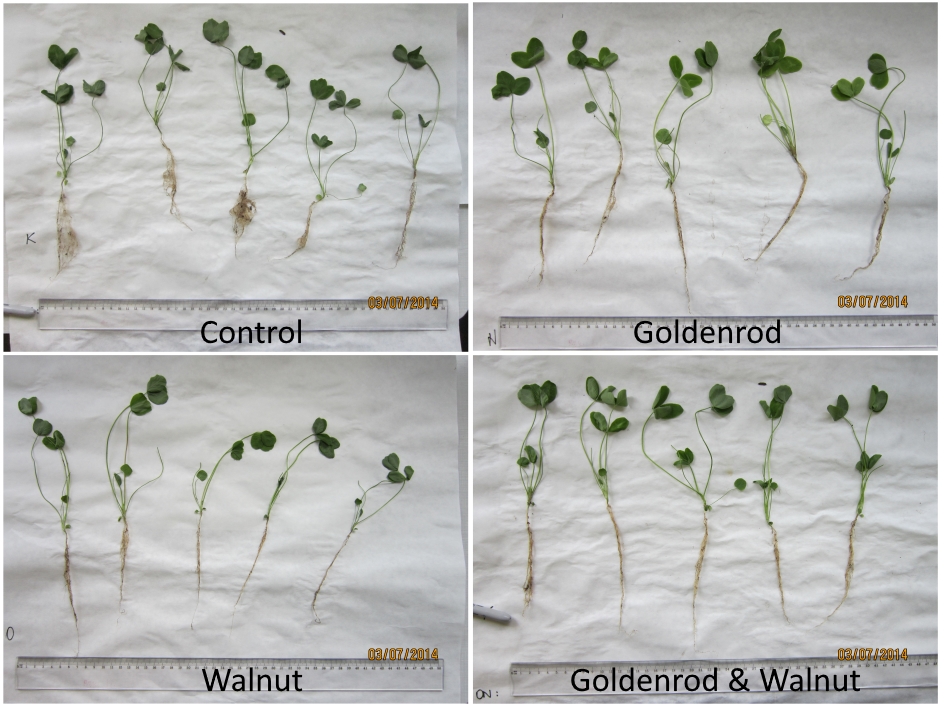


Figure S7. The effect of treatment on the morphology of *Trifolium repens* seedlings growing in flowerpots. Treatments were water (control), extracts from goldenrod, extracts from walnut, and mixed extracts from both the species. Photos by Zuzanna Zagrodzka.

**Literature**

Bączek-Kwinta, R. (2017). Swailing affects seed germination of plants of European bio-and agricenosis in a different way. Open Life Sciences, 12, 62-75

Bilger, W. & Björkman, O. (1991). Temperature-dependence of violaxanthin deepoxidationde-epoxidation and nonphotochemicalnon-photochemical fluorescence quenching in intact leaves of *Gossypium hirsutum* L. and *Malva parviflora* L. Planta, 184, 226-234

Borek, M., Bączek-Kwinta, R. & Rapacz, M. (2016). Photosynthetic activity of variegated leaves of *Coleus* *x* *hybridus* Hort. cultivars characterised by chlorophyll fluorescence techniques. Photosynthetica, 54, 331-339

Carrion, J.S. & Sanchez-Gomez, P. (1992). Palynological data in support of the survival of Walnut (Juglans regia L.) in the western Mediterranean area during last glacial times. J. Biogeogr., 19, 623-630

Ercisli, S., Esitken, A., Turkkal, C. & Orhan, E. (2005). The allelopathic effects of juglone and Walnut leaf extracts on yield, growth, chemical and PNE compositions of strawberry cv. Fern. Plant, Soil and Environment, 6, 283-287

Harborne, J.B. (1977). Flavonoid profiles in the Compositae. Pages 359-384 in Heywood YH, Harborne JB, Turner BL, eds. The biology and chemistry of the Compositae. Academic Press, New York. I 189 pp

Harrison, X.A., Donaldson, L., Correa-Cano, M.E., Evans, J., Fisher, D.N., Goodwin, C.E.D., Robinson, B.S., Hodgson, D.J. & Inger, R. (2018). A brief introduction to mixed effects modelling and multi-model inference in ecology. PeerJ, 6, e4794. doi:[10.7717/peerj.4794](https://doi.org/10.7717/peerj.4794)

Hegnauer, R. (1977). The chemistry of the Compositae. Pages 283-336 in Heywood YH, Harborne JB, Turner BL, eds. The biology and chemistry of the Compositae. Academic Press, New York. I 189 pp

Herz, W. (1977). Astereae- chemical review. Pages 567-576 in Heywood YH, Harborne JB, Turner BL, eds. The biology and chemistry of the Compositae. Academic Press, New York. I 189 pp

Huntley, B. & Birks, H.J.B. (1983). An atlas of past and present pollen maps for Europe: 0-13,000 years Ago. Cambridge University Press, NY

Pastorello, E.A., Farioli, L., & Pravettoni, V. (2004). Lipid transfer protein and vicilin are important Walnut allergens in patients not allergic to pollen. J. Allergy Clin. Immunol., 114, 908-914

Ponder, F. & Tadros, S.H. (1985). Juglone concentration in soil beneath Black Walnut interplanted with nitrogen-fixing species. Journal of Chemical Ecology, 11, 937-942

R Core Team (2017). R: A language and environment for statistical computing. URL http://www.R-project.org/. R Foundation for Statistical Computing, Vienna, Austria

Schreiber, U., Schliwa, U. & Bilger, W. (1986). Continuous recording of photochemical and non-photochemical chlorophyll fluorescence quenching with a new type of modulation fluorimeter. Photosynthesis Research, 10, 51-62

Vander Wall, S.B. (2001). The evolutionary ecology of nut dispersal. Bot. Rev., 67, 74-117

Zohary, D. & Hopf, M. (1988). Domestication of plants in the old world. 1st edn. Oxford University Press, Oxford
